# Editorial Notes

**Published:** 1908-06

**Authors:** 


### Editorial Notes.

**The Coming University.**

Some of the essential principles in the preliminary steps towards the basis of formation of a Bristol University are under active consideration, and there is every reason to believe that everything will be satisfactorily settled very shortly. We hope there will be a liberal response on the part of the public who realise the value and importance of such a University to Bristol as a whole and to the neighbourhood of Bristol, in addition to the enormous assistance such an institution must have on the advance of higher education in the West of England.

It is hardly thinkable that Bristol will remain long without its University. In the past this city has sometimes allowed its opportunities to lapse for want of a little enterprise and initiative. At tremendous cost it has determined to make up leeway in its commercial aspects by the expenditure of nearly three million pounds for its new docks. It would be lamentable if similar policy with regard to educational matters led Bristol to allow this opportunity for acquiring University status to lapse.

The only question apparently is whether the coming University will be a strong union of available forces or somewhat less strong from lack of complete union.

We are glad to see that an effort is being made to raise sufficient funds to endow a Chair of Anatomy in memory of the late Dr. E. Markham Skerritt. His work in connection with the school is too well known to require any notice here; and we cannot imagine any happier way of perpetuating the memory of one who did so much for the Faculty of Medicine in University College, Bristol.

**Tuberculosis.**

Dr. Bulstrode's supplement on "Sanatoria for Consumptives, and certain other aspects of the Tuberculosis Question."

Winsley Sanatorium Third Annual Report, 1907.

The various papers bearing on tuberculosis published in this number, as also in a recent number of the *Practitioner* (May, 1908), are giving the subject a degree of prominence which only its great importance can justify.

Sir Dyce Duckworth, in his address to the Faculty of Medicine in Paris last February, emphasised the view that the great advances in laboratory work have directed especial attention to the seed, but it is all the more necessary that the soil, as distinct from the seed, should continue to have the fullest consideration. The clinical physician cannot give place entirely to the pathologist and bacteriologist, whose work, however valuable it may be, is only a part of the general management of the patient. Questions relating to the bacillus can only form a small part of the treatment of tubercular diseases, and hence the sanatorium should, and doubtless will, continue to take the leading place in the treatment of tubercular diseases. Questions of climate are dropping into the background, as it is now generally admitted that there is truth in the aphorism of Solly that "without hygiene climate is as sounding brass or a tinkling cymbal." It has been estimated by Flexner that at least 90 per cent. of human beings are at some time of their lives infected with the tuberculous virus, and the history of the coloured races in America—negroes and Indians—more recently also the liability to phthisis of the Irish race in America, shows how the neglect of hygienic conditions must in these cases lead to the extinction of tribes and races of mankind. The tendency is born in everything, and it is admitted that as the task of the extirpation of all tubercle bacilli is impossible, it becomes, therefore, indispensable to so strengthen and harden the body that the absorbed germs cannot take hold upon it. That is what our hygienic methods are attempting to do, and our sanatoria are going a stage further in endeavouring to stamp out the malady where it has already obtained a hold on the subject. It is admitted that early tuberculosis is a simple condition, easily cured; that the advanced disease is a complex one, cured with difficulty.

It cannot be admitted that fresh air acts as a specific poison to the bacillus: it may give better tone and improve the resisting power, and so bring about "Nature's cure" without any adventitious aids; it is therefore at the basis of sanatorium methods. But the clinical cure depends upon the intelligence of the physician in charge, and the co-operative response of the patient, who must himself "work out his own salvation" by every means in his power. The endeavour to eradicate the disease has been well commenced, but it will not be accomplished in the near future. Whilst we know that 80 per cent. may recover if detected early, yet our results must inevitably fall short of this because of the difficulty of early detection. The patient does not know that he is consumptive till the disease has obtained a secure footing, and is often well advanced. In spite of the endeavours of the hygienic preachers—we know the facts, and the public are almost weary of much preaching—yet the appalling waste goes on.

Formerly the physician did his best to conceal his diagnosis of the fatal malady; the patient was kept in ignorance, even if the physician knew. The reluctance to accept the responsibility of a diagnosis was formerly justifiable, but it is not so now. The physician must not shirk the responsibility, a grave one, inasmuch as the curability of the patient depends on his acumen. We must find the consumptive early—early diagnosis is the transcendent requisite. Now the newer methods, the ophthalmo-reaction of Calmette, the methods of X-ray diagnosis, and all other means must be used to discover the disease at a date anterior to the presence of well-marked physical signs.

It is to be hoped that the simple hygienic treatment by sanatorium life will in the near future receive powerful assistance by means of tuberculin methods. Dr. Munro's paper (page 97) shows that under opsonic guidance the injection of tuberculin is not only safe but sometimes efficient. Evidence in this direction is accumulating day by day, and our sanatoria should in the near future give much better results than they have done hitherto; but for this funds are greatly needed, and we must appeal for a more sympathetic consideration from the philanthropic public, and a greatly increased financial support.

The following extract from the very full and detailed report on sanatoria, recently published by Dr. H. Timbrell Bulstrode on behalf of the Local Government Board, is worthy of careful consideration. He states that :—

> " In order to obtain the best results as regards working capacity, two things are necessary. One is that the case shall be suitable for treatment, and that the patient shall remain at the sanatorium long enough to prove by actual experience that he is competent to work : the other is that there shall be a period of after-care, during which the patient may gradually become accustomed to full work, and may, if necessary, learn the means of gaining a livelihood in the manner best suited for the maintenance of his health."

The general inference from a perusal of this report is that in the handling of tuberculosis among the working classes we are lamentably behind Germany, where the compulsory insurance system occupies a foremost place in the anti-tuberculous crusade, and is one of the most important agents in the production of a remarkable fall in the death rate from tuberculosis. Probably Bismarck did not foresee all the results of the insurance legislation of 1884, but it is most satisfactory that the results which have been obtained have been far in excess of anything which was expected.

We may, however, claim that the medical profession in this country has not been behindhand in endeavouring to induce the philanthropist and the public to follow in the footsteps of the German pioneers.

**"Confessio Medici."**

THE author of *Confessio Medici* (Macmillan) writes a highly-entertaining book on the vocation of medicine. We quote the following as a sample of his views :—

" The setting of the life of a hospital—the buildings and such works of art as the hospital may possess—are shown to every visitor. But to observe its mind the visitor need not go farther than the hospital garden, that quiet place which is no more like the outside world than home is like a music-hall. For the *genius loci* loves the open air, and the hospital garden is his sacred grove. And it shall be summer-time, with patients lying under the shade of the plane-trees, and nurses bringing tea to them; and students, bare-headed, playing tennis, or talking over the day's affairs, or just doing nothing. Of itself it may not be much of a garden, but we admire it as an open space, a great well of light and air, a shaft of health sunk into diseased and injured London. This pleasant, old-fashioned quadrangle, blessed with sunshine and silence, and an excellent view of the sky, is the centre of the hospital life, and a visitor loitering here will see that we are a brotherhood, and that the patients are our guests. Every hospital is a charity, but there is a difference between charity and hospitality. They who give money to hospitals are charitable; we who have the spending of it are hospitable, and of course it is we who get the fun out of the money. And we spend it well, entertaining in good style our innumerable guests. . . . Let us hold fast to the unity of hospital life, and to our bounden duty to the spirit of the place. . . . No profession gives to its students such a good introduction to things as they are."

**Territorial Army.**

At a meeting of the profession, held in the Medical Library on March 20th to inaugurate the opening of the Public Health Laboratory of University College, Lt.-Col. Firth, R.A.M.C., spoke at some length on the subject of the "Sanitary Service of the Territorial Army." From his remarks we gather that the principal medical officer of an area would have on his head-quarters' staff a sanitary medical officer, who will advise him on all questions of sanitation in times of peace and war. In peace at training camps each regimental surgeon is responsible to his colonel for the sanitary condition of his camp, and for carrying out general orders issued by the head-quarters' sanitary officer. The Field Ambulance officers are only responsible for the condition of their own camp. In time of war, or when the troops are embodied, other sanitary medical officers—probably drawn from the medical officers of health of the counties comprising the area—will be pressed into the service, and serve in their branch just as the *en suite* physicians and surgeons serve who are connected with the General Hospital.

We venture to suggest that this plan is inadequate. The regimental medical officer is a busy man, and would have insufficient time, and perhaps experience, to cope with any serious sanitary defect, such as water contamination, unfit camp surroundings, and epidemics; and we think that where a division, or even a brigade, is massed in camps, medical officers of health ought to be asked to be in a position of responsibility for the health of the troops.

The South-Midland area, to which Bristol belongs, comprises the counties of Gloucester, Warwick, Worcester, Oxford, Berkshire and Buckingham, and the head-quarters are at Birmingham. It seems to us a waste of good material for the division in camp not to have the advantage of the excellent and energetic medical officers of health that there are in Bristol and those counties.

